# Interference Screw vs. Suture Anchor Fixation for Open Subpectoral Biceps Tenodesis: Does it Matter?

**DOI:** 10.1186/1471-2474-9-121

**Published:** 2008-09-15

**Authors:** Peter J Millett, Brett Sanders, Reuben Gobezie, Sepp Braun, Jon JP Warner

**Affiliations:** 1 Steadman Hawkins Clinic & Steadman Hawkins Research Foundation, 181 West Meadow Drive, Suite 1000, Vail, CO. USA; 2 Harvard Shoulder Service, Brigham & Women's Hospital, 75 Francis St, Boston, MA. USA; 3 The Case Shoulder & Elbow Service, Case Western Reserve University School of Medicine, Cleveland, OH. USA; 4 Presented at the American Orthopaedic Society for Sports Medicine 2006 Annual Meeting. Hershey PA

## Abstract

**Background:**

Bioabsorbable interference screw fixation has superior biomechanical properties compared to suture anchor fixation for biceps tenodesis. However, it is unknown whether fixation technique influences clinical results.

**Hypothesis:**

We hypothesize that subpectoral interference screw fixation offers relevant clinical advantages over suture anchor fixation for biceps tenodesis.

**Study Design:**

Case Series.

**Methods:**

We performed a retrospective review of a consecutive series of 88 patients receiving open subpectoral biceps tenodesis with either interference screw fixation (34 patients) or suture anchor fixation (54 patients). Average follow up was 13 months. Outcomes included Visual Analogue Pain Scale (0–10), ASES score, modified Constant score, pain at the tenodesis site, failure of fixation, cosmesis, deformity (popeye) and complications.

**Results:**

There were no failures of fixation in this study. All patients showed significant improvement between their preoperative and postoperative status with regard to pain, ASES score, and abbreviated modified Constant scores. When comparing IF screw versus anchor outcomes, there was no statistical significance difference for VAS (p = 0.4), ASES score (p = 0.2), and modified Constant score (P = 0.09). One patient (3%) treated with IF screw complained of persistent bicipital groove tenderness, versus four patients (7%) in the SA group (nonsignificant).

**Conclusion:**

Subpectoral biceps tenodesis reliably relieves pain and improves function. There was no statistically significant difference in the outcomes studied between the two fixation techniques. Residual pain at the site of tenodesis may be an issue when suture anchors are used in the subpectoral location.

## Background

The long head of the biceps tendon is frequently a significant source of pain in the shoulder as a result of pathology related to instability, trauma, or inflammation.[1-3] When conservative measures at treatment have failed, surgical tenotomy or tenodesis are options for treatment. Many authors believe that tenodesis decreases the risk of cosmetic deformity, strength loss and cramping inherent to biceps tenotomy alone.[4-6] Biceps tenodesis was originally described by Gilcreest, [7] and has been performed by a variety of surgical methods in the ensuing years. Among these methods, differences center around open versus arthroscopic techniques, location of tenodesis and method of fixation. Although many authors report good short term outcomes with proximal techniques, long term results have been less encouraging. [8,9] Tenodesis of the biceps tendon proximal to or within the biceps groove does not address residual synovium in this area which could act as a persistent pain generator. [10-12] As a result, arthroscopically assisted open subpectoral tenodesis [13,14] has evolved as a viable method which allows assessment and treatment of intra-articular pathology, as well as a cosmetically acceptable tenodesis at the distal portion of the intertubercular groove. This method also has the advantage of technical simplicity and utilization of an intermuscular interval for surgical dissection.

There are a variety of tenodesis methods available to the surgeon, including fixation through a bone tunnel, [2] the keyhole method, [15] soft tissue tenodesis to the rotator interval or conjoint tendon, [16,17] interference screw fixation [10,11,18] and suture anchors. [4] The optimal method should be simple, fast, and afford adequate initial fixation strength to maintain the appropriate length-tension relationship of the biceps tendon during rehabilitation until healing occurs. Biomechanical stability of different methods of tenodesis have been studied by several authors. [6,19-21] Mazzocca et al. found that suture anchors (164 N) and IF screw fixation (252 N) were superior to bone tunnel fixation with regard to displacement after cyclic loading, but were unable to show statistical differences in load to failure between the constructs. [6] Ozlay et al. demonstrated in a sheep model that interference screw fixation (243 N) was superior to bone tunnel (229 N), suture anchor (129 N) and keyhole technique (101 N). [19] Richards and Burkhart's cadaveric data revealed that load to failure of a biotenodesis screw (233 N) was superior to a double suture anchor technique (135 N). [20]

The authors believe that subpectoral tenodesis offers the advantages of technical simplicity, healing of tendon within bone and tenodesis in a distal location free from synovium and residual tendon disease. Whether the type of fixation has an additional benefit remains unknown. Although the biotenodesis screw offers a theoretical advantage in increased initial fixation strength, presumably decreasing the risk of early failure during rehabilitation, this has not been demonstrated to be a relevant advantage clinically. We hypothesize that the smoother tendon-bone transition and intramedullary location of the interference screw (IF screw) may improve postoperative results compared to suture anchors. The goal of the present study therefore is (1) to evaluate the results of subpectoral biceps tenodesis in a large cohort of patients and (2) to evaluate whether tenodesis with an IF Screw technique offers clinically relevant advantages over tenodesis with suture anchors (SA).

## Methods

A retrospective review was performed on 88 consecutive patients receiving an arthroscopically assisted, open subpectoral tenodesis from October 2001 to March of 2005 by the two senior authors. This retrospective study obtained IRB approval from the Brigham and Women's Hospital, Boston, MA. The study was performed with written consent of all participating patients. All of the patients were tenodesed in the inferior aspect of the bicipital groove at the lower border of the pectoralis major tendon. Each patient received biceps fixation with either interference screw fixation (34 patients) or suture anchor fixation (54 patients). Forty-eight of the 54 had single suture anchor fixation, while the remainder had double suture anchors. Nineteen of the IF screw group had 8 × 12 mm IF screws, with the remainder of the screws ranging from 5.5 to 7 mm in diameter. The tenodeses were performed for the clinical diagnoses of biceps tenosynovitis, partial tear > 50% of the tendon, or biceps tendon subluxation. These diagnoses were confirmed at time of arthroscopic evaluation of the glenohumeral joint. Thirty-nine of these were work related injuries and 8 patients in the total cohort were revision tenodeses. Eighty-two patients had another associated procedure at the time of surgery (12 AC joint resection, 64 rotator cuff repair, 41 acromioplasties, 8 capsular reconstruction). Average follow up was 13 months (range 3 to 25 months).

The study cohort consisted of 31 females and 57 males with a mean age of 51 (range 22 to 77). Because cosmesis was also considered an endpoint, we calculated the mean BMI for the study group which was 27. This is designated as overweight by National Institute of Health criteria. [22] There were 33 left and 55 right shoulders. Thirty-nine were work related.

Outcome variables studied were pain on a VAS Scale of 0–10, (0 = no pain, 10 = worst pain), American Shoulder and Elbow Surgeons Score (ASES) (0–100 point scale), and abbreviated modified Constant Score without strength testing(maximum score of 75). [23,24]

Length of surgical time and surgical complications were evaluated. Popeye deformity or loosening of the fixation device was considered failures. Additionally, subjects were evaluated both preoperatively and postoperatively for tenderness in the proximal intertubercular groove and at the site of tenodesis, as well as for the presence of a "popeye" deformity. Deformity was either evaluated clinically at follow up by the physician or the patients were asked questions about cosmetic concerns or appearance of brachium and any spasms of the biceps by phone interview. The subjects were also questioned regarding subjective postoperative cosmetic concerns, as well as pain or spasms in the biceps muscle belly. At the latest time of follow up, subjects were contacted by phone and asked the following questions: Is your shoulder better, the same, or worse than before surgery? Are you glad you had the surgery? Would you have surgery again? Are you back to normal activities?

## Results

There were no failures of fixation and no complications postoperatively in the entire study group. Five patients complained of persistent bicipital groove tenderness (1 IF and 4 SA group). There were no 'popeye' deformities or complaints of cosmetic concern. Two patients in the IF and two in the SA complained of persistent spasm in the biceps.

Overall, there were no statistically significant differences between the two types of fixation with respect to the outcomes evaluated. Table 1 shows pre- and postoperative patient data.

**Table 1:** Interference screw versus suture anchor data.

	Interference Screw	Suture Anchor
Length of Surgery	1.76 hrs 95% Confidence Interval [1.55, 1.97]	1.91 hrs 95% Confidence Interval [1.74, 2.09]
Preoperative Grove Tenderness	32/34	49/54
VAS pain Preoperative (scale 0 = no pain, 10 = worst)	9	9
Preoperative ASES (0–100)	30	27
Preoperative Modified Constant	30	28
Complications	0/34	0/54
Postoperative Aesthetic Concerns	0/34	0/54
Postoperative Grove Tenderness	1/34	4/54
Postoperative Deformity of Biceps	0/34	0/54
Postoperative Spasm or Biceps Pain	2/34	2/54
Postoperative VAS pain (scale 0 = no pain, 10 = worst)	2.5	2.6
Postoperative ASES (0–100)	74	77
Postoperative Modified Constant	57	60

### Pain

The median preoperative pain score was 9 (IQR = 8–10, full range = 4–10) and the median postoperative score was 2 (P < 0.0001, Wilcoxon signed-ranks test) for the entire group. Both IF Screw and suture anchor technique had preoperative and postoperative mean pain scores of 9 and 2, respectively.

### Subjective Outcome Measures

Subjective outcome measurement data is available for 20 patients of the IF screw group. Postoperatively, (95%) report that their shoulder is allover better or the same as preoperatively. One (5%) said his shoulder was worse (P = .24). And another patient did not come back to his normal activities.

In the suture anchor group, 35 responded when asked is your condition better, same or worse? For 28 (80%) their shoulder was better or same postoperatively. Seven (20%) reported that their shoulder was overall worse postoperatively. Twenty-nine (82%) patients were glad to have had surgery. One patient answered, that he was glad that he had the surgery and would undergo the procedure again although his shoulder was not better after the procedure. Thirty (86%) patients in this group came back to their normal activities. Subjective outcome data is presented in Table 2.

**Table 2:** Interference screw versus suture anchor patient outcome questions.

	Interference Screw		Suture Anchor		P value
Response	Yes	No	Yes	No	
Shoulder is better/same?	19	1	28	7	0.2
Glad you had the surgery?	15	5	29	6	0.5
Would you have the surgery again?	16	4	28	7	1.0
Are you back to your normal activities?	19	1	30	5	0.3

### Score Outcome Data

Pre- and postoperative score outcome data has been collected on all patients.

The preoperative and postoperative ASES scores changed significantly from 28 (range 7 – 58) to 76 (range 30 – 98) (P < 0.0001, paired *t*-test) for the entire group. ASES scores were 30 (range 8 – 45) preoperatively and 74 (range 30 – 98) post operatively for the IF screw. For the suture anchor group, pre and postoperative ASES scores were 27 (range 10 – 58) and 77, (range 37 – 97) respectively. There was no statistically significant difference between the suture anchor and IF screw group (P = 0.2, ANOVA and Wilcoxson Rank Sum Test), but both showed a significant score improvement (both P < 0.0001, paired *t*-test).

The preoperative and postoperative modified Constant scores were 29 (range 8 – 50) and 59 (range 25 – 71) respectively for the entire group (P < 0.0001, paired t-test). The Preoperative and postoperative modified Constant scores were 30 (range 8 – 45) and 57 (range 25 – 71), respectively for the IF screw Group. The Preoperative and post operative modified Constant scores were 28 (range 10 – 50) and 60 (range 27 – 71), respectively, for the suture anchor group. There was no statistically significant difference between the SA and IF screw group (P = 0.0872, Wilcoxon Rank Sum two-sided Test), but both showed a significant score improvement in their group (both P < 0.0001, paired *t*-test).

## Discussion

In this study, a subpectoral biceps tenodesis performed with either a biceps tenodesis IF screw or suture anchors were found to be equally reliable in relieving pain and improving function by validated outcome measures. There were no statistically significant differences in the outcomes studied between the two fixation techniques. Cosmetic concerns and deformity are no problem with either technique. Operative times were similar and in both groups there were no complications.

Although there are a multitude of papers in the literature describing subpectoral tenodesis techniques, there are few published reports on results. Biomechanical data from previous authors implies that a biotenodesis screw is mechanically stronger in load to ultimate failure in cadaver models. [6,19,20] However, this finding does not appear to be clinically relevant in our series since there were no deformities noted in either group. One confounding variable in our series was that our average patient was overweight by BMI criteria, which may lessen the perception of cosmetic deformity by obscuring the distally retracted long head of the biceps. But, two patients in the SA group and two in the IF screw group (workers compensation related and revision surgery) complained of post operative spasms. With regard to spasm in the biceps, both techniques in our series were equivalent. Persistent spasms may be a result of inappropriate tension of the biceps at the time of surgery, which is a risk regardless of technique. Care should be taken to note the resting length of the tendon and try to re-establish the correct length. With this technique of subpectoral tenodesis, the tenodesis should take place approximately 1–2 cm proximal to the musculotendinous junction.

Based on the clinical finding of residual pain at the tenodesis site in some postoperative patients, we investigated the presence of pain at the tenodesis site with two methods of biceps fixation. We hypothesized that a biotenodesis screw provided a smoother transition of tendon to bone without the presence of residual prominent suture material under the pectoralis major tendon, theoretically generating less mechanical irritation in this area. Although there was a trend toward persistent pain in the groove with the use of suture anchors (7% versus 3%), this finding was not statistically significant with the numbers available in our study and at the time of our follow up. In contrast, some reports of synovitis or sterile inflammatory response have been noted [25-28] with the use of bioabsorbable polylactic acid devices up to 5 years after implantation. Although this phenomenon has been reported extra and intraarticularly, we are aware of no reports of its occurrence in the subpectoral location. It remains to be seen if foreign body reaction to a bioabsorbable screw can account for persistent pain at the tenodesis site, although data from this study do not support such a phenomenon. Historical studies by Becker and Cofield published in 1989 had long term data (average 13 years) that showed 50% of tenodesis fail.  Dines et al published in 1982 a study that showed about 30% failure at 3 years. [8,9]

We questioned our patients at a mean postoperative time of 13 months about their global subjective perception of the success or failure of the surgery. Eighty percent of the IF screw group responded that they were improved and were glad to have had the surgery, as opposed to 75% of suture anchor group. Ninety-five percent of the IF screw group reported that they had returned to normal daily activities, while only 86% of the SA group had returned to daily activities. The desire to repeat the surgery in retrospect was similar between the two groups. There was a trend toward a more positive subjective result with the IF screw group. However, due to the coexistence of other pathology, it is difficult to infer a direct correlation of global success with biceps fixation method alone. Since this was a retrospective study with many confounding variables, there may be bias which could influence our findings. Further study is needed on clinical outcomes to ascertain the best method of fixation and the long term results of biceps tenodesis.

## Conclusion

We found that subpectoral biceps tenodesis provided a reliable means for treating pathology of the biceps with no cosmetic deformities and significant alleviation of pain. There was no statistical difference in standardized outcome measures, postoperative subjective patient outcome, deformity, or pain at the fixation site when IF screw fixation was compared retrospectively to suture anchor fixation for subpectoral biceps tenodesis. The results of this study underscore an important point that most results reported on biceps tenodesis showing good results are short term.

## Competing interests

Financial competing interests:

Peter J. Millett discloses a financial relationship with Arthrex but it is unrelated to this manuscript. All authors declare that they have no competing interests.

## Authors' contributions

All authors made substantive intellectual, conception and design contributions to this study. All authors took part in data acquisition, analysis, interpretation, drafting and gave final approval of the manuscript.

BS was taking part in study design, data acquisition, analysis, interpretation, drafting and gave final approval of the manuscript.

RB was taking part in study design, data acquisition, analysis, interpretation, drafting and gave final approval of the manuscript.

SB made substantial contribution to data analysis and interpretation, revised the manuscript critically and gave approval of the final version.

JJPW performed surgeries, was taking part in study design, data acquisition, analysis, interpretation, drafting and gave final approval of the manuscript.

PJM was the primary investigator. He initiated and conceptually designed the study; he performed a major part of the surgeries and took part in the data collection. He was participating in data analysis, interpretation, drafting and gave final approval of the manuscript.

## Pre-publication history

The pre-publication history for this paper can be accessed here:


